# The influence of cognitive function on adherence to breast cancer screening among older American women

**DOI:** 10.1093/geroni/igab046.3534

**Published:** 2021-12-17

**Authors:** Chien-Ching Li, Fritzi Flores, Alicia K Matthews, Bryan James, Raj Shah

**Affiliations:** 1 Rush University, Rush University, Illinois, United States; 2 University of Illinois at UIC, Chicago, Illinois, United States; 3 Rush University, Chicago, Illinois, United States; 4 Rush, Rush University Medical Center, Illinois, United States

## Abstract

Cognitive decline and impairment among older adults have become an important public health issue. Previous research shows older women have a greater prevalence of Alzheimer's disease than Men. Among women, breast cancer is one of the most common types of cancer. Over half of breast cancer deaths occur in women aged 65 and older. Therefore, early detection of breast cancer through mammogram screening is important among older women. This study aimed to examine the influence of cognitive function on adherence to mammogram breast cancer screening among older American women aged 65 and older. Data from the Health and Retirement Study (2012-2016) was obtained and analyzed. The independent variable of the study was cognitive function (normal, not normal). Adherence to mammogram (low, moderate, high) was the dependent variable. Multinomial regression was performed to examine the association between cognitive function and adherence to mammogram after controlling for demographic covariates. In the study, 33.3% of respondents had impaired cognitive function and 21.7% showed low adherence to mammogram screening. Regression results found that older women with impaired cognitive function were more likely to be in low adherence group (OR=1.30, p=0.01) or moderate adherence group (OR=1.47, p<0.001) relatively to be in high adherence group compared to older women with normal cognitive function. The development and implementation of interventions are needed for reducing barriers to accessing cancer screening services in high-risk vulnerable populations. This submission is considered late-breaking research because study findings were obtained from a recently completed student's master's project.

